# Increasing Referrals to a YMCA-Based Diabetes Prevention Program: Effects of Electronic Referral System Modification and Provider Education in Federally Qualified Health Centers

**DOI:** 10.5888/pcd12.150294

**Published:** 2015-11-05

**Authors:** Earle C. Chambers, Judith Wylie-Rosett, Arthur E. Blank, Judy Ouziel, Nicole Hollingsworth, Rachael W. Riley, Peter A. Selwyn

**Affiliations:** Author Affiliations: Judith Wylie-Rosett, Department of Epidemiology and Population Health, Albert Einstein College of Medicine, Bronx, New York; Arthur E. Blank, Peter A. Selwyn, Department of Family and Social Medicine, Albert Einstein College of Medicine, Bronx, New York; Judy Ouziel, YMCA Diabetes Prevention Program, Activate America, New York, New York; Nicole Hollingsworth, Rachael W. Riley, Office Of Community and Population Health, Montefiore Medical Center, Bronx, New York.

## Abstract

**Introduction:**

The Diabetes Prevention Program has been translated to community settings with varying success. Although primary care referrals are used for identifying and enrolling eligible patients in the Diabetes Prevention Program, little is known about the effects of strategies to facilitate and sustain eligible patient referrals using electronic health record systems.

**Methods:**

To facilitate and sustain patient referrals, a modification to the electronic health record system was made and combined with provider education in 6 federally qualified health centers in the Bronx, New York. Referral data from April 2012 through November 2014 were analyzed using segmented regression analysis.

**Results:**

Patient referrals increased significantly after the modification of the electronic health record system and implementation of the provider education intervention. Before the electronic system modification, 0 to 2 patients were referred per month. During the following year (September 2013 through August 2014), which included the provider education intervention, referrals increased to 1 to 9 per month and continued to increase to 5 to 11 per month from September through November 2014.

**Conclusions:**

Modification of an electronic health record system coupled with a provider education intervention shows promise as a strategy to identify and refer eligible patients to community-based Diabetes Prevention Programs. Further refinement of the electronic system for facilitating referrals and follow-up of eligible patients should be explored.

## Introduction

The Centers for Disease Control and Prevention (CDC) estimates that 25.8 million people in the United States have diabetes and that another 79 million people have prediabetes (1). With the increased cost for treating diagnosed diabetes (2), preventing diabetes among people with prediabetes should be a priority. The Diabetes Prevention Program (DPP) was a randomized trial consisting mostly of women (68%) that compared a lifestyle modification program to metformin (drug therapy) and placebo. Results showed that the lifestyle modification program decreased the risk of type 2 diabetes by 58% compared with a 31% decrease among those on metformin (3). The DPP lifestyle 16-session core curriculum (4) and goals for weight loss and physical activity were developed with the intent of translating the program into community-based settings (5,6). The CDC-sponsored National DPP initiative and the Diabetes Prevention and Control Alliance were established to create the infrastructure to support the upscaling of DPP into community-based settings (7,8).

The YDPP (YMCA Diabetes Prevention Program) has been widely implemented and evaluated in community settings (8–12). The New York State YDPP weight loss results were similar to those of the original study (approximately half of participants achieved 5% weight loss); however, Medicaid recipients constituted only 6.7% of the participants, and few participants were black or Hispanic (13). Identifying eligible patients in a clinical setting and referring them to community-based DPPs may help increase participation in DPP programs in high-risk communities (14). Although referrals through primary care have been successful for identifying and enrolling eligible patients in YDPP (8), studies show that less than 10% of primary care providers refer their overweight or obese patients to nutritional specialists or weight-loss programs (15–17). Providers often cite difficulty in the process of referring patients to available programs as a barrier to referring patients for behavioral interventions (16). Little is known about the effects of reducing the referral burden by modifying the electronic health record (EHR) system and providing staff education. 

In our target clinics, few, if any, patients were being referred to local YDPP programs. This article reports the results of modifying the EHR system for ease of patient referral combined with a provider education intervention to increase and sustain clinic-based YDPP referrals over time in federally qualified health centers (FQHCs) in the Bronx, New York.

## Methods

### Data sources

Bronx CATCH (Collective Action to Transform Community Health) is a partnership between Montefiore Medical Center (MMC) and designated FQHCs, the Bronx District Public Health Office of the New York City Department of Health and Mental Hygiene (DOHMH), and other community-based organizations that was established in 2010 and devoted to health and well-being in the Bronx. CATCH prioritized diabetes prevention strategies, because the prevalence of diagnosed diabetes in the affiliated FQHCs is approximately 19%, which is nearly 10% higher than the national prevalence (18). YDPP was chosen as an evidence-based intervention that built on existing collaborations between CATCH partners. The data presented in this study were collected to evaluate ongoing referrals made by MMC FQHCs under the CATCH initiative to YDPP from April 2012 through November 2014. The institutional review board of MMC approved the activities associated with the data presented in this article.

Six CATCH-affiliated FQHCs were selected as the pilot locations for this YDPP intervention. These FQHCs are located throughout the Bronx, servicing an area in which more than 70% of the population and clinic patients are black or Hispanic (19,20). For patients to be eligible for YDPP, they must meet the criteria set by YDPP, which include being aged 18 or older, being without a previous diagnosis of diabetes, and having a hemoglobin A1c value between 5.7% and 6.4% (fasting plasma glucose of 100–125 mg/dL or 2-hour plasma glucose of 140–199 mg/dL can also be used). The patient must also be overweight or obese, defined as a body mass index (BMI) of 25 kg/m^2^ or more (calculated from weight divided by height squared; BMI ≥22 if Asian). 

To refer a patient, the provider clicks the “YMCA DPP” referral order in the EHR, and the form autopopulates and prints with patient contact and eligibility information, as well as clinic referral information, requiring only patient and provider signatures. The form is sent by fax to the YMCA, where the patient’s information is entered and stored in an electronic database, and the patient is subsequently contacted by YMCA staff to enroll in YDPP. Patient referral to the YDPP by a health care provider is part of a patient’s normal clinical care and requires patient consent for information to be sent to YDPP staff. Data about patient referrals to the YDPP are captured by the YMCA and reported monthly to MMC. Seven months after implementing this new electronic system of preparing the YDPP referral form, a formal presentation was made to the medical directors and clinical staff at each of the FQHCs informing them of this new system and encouraging them to refer eligible patients using this system.

### EHR lead-in and provider education period

In September 2013, a new electronic referral to YDPP was introduced into the EHR system at MMC. An order form to electronically print the referral for YDPP and capture the patient, provider, clinic, and time of the referral was designed and implemented for ease of use and sustainability of referrals. The medical directors and clinical staff received an overview presentation and instructional guide for providers about the YDPP referral process during a CATCH meeting for all of the FQHC leaders in April 2014. A subsequent YDPP program information presentation for the FQHC leaders was conducted during the July 2014 CATCH meeting.

### Statistical analysis

Segmented regression analysis was used to determine whether there was an increase in the number of referrals to the YDPP in our target clinics after the lead-in EHR period and the provider education intervention and whether that change was maintained after the intervention period. Segmented regression is a robust analytic technique ideal for analyzing data from a quasi-experimental approach or natural experiment in which preintervention and postintervention data are available (ie, an interrupted time series) but data are not the result of a clinical trial design (21,22). The advantage of segmented regression analysis is the ability to identify and assess trends in the data before and after an intervention in which random assignment was not done or not possible and there was no control group. This type of analysis is becoming more common in health research and evaluation (21–23).

Segmented regression provides 3 pieces of information that quantifies patient referrals before, during, and after the interventions (Figure). A regression line is fit to the data that are collected before the intervention (PreSlope) and a separate line is fit to the data collected after the intervention (PostSlope). The PreSlope estimate shows the referral trend before the intervention, and the PostSlope estimate shows whether there was a sustained change (ie, increase, decrease, or no change) in referrals after the intervention. The third parameter estimate calculated measures the change in level of the preregression and postregression lines at the point of the intervention (Int). This estimate captures the relative increase or drop in referrals following the intervention point. The following regression equation was used in this study to assess the EHR lead-in and the provider education intervention:

Υ_t_ = β_0_ + β_1_(PreSlope1_t_) + β_2_(Int1_t_) + β_3_(PostSlope1_t_) + β_4_(Int2_t_) + β_5_(PostSlope2_t_) + *e*_t_

where Υ_t_ is the dependent variable, number of referrals, at time t. β_0_ is the intercept term indicating the baseline referrals at the beginning of the study period; β_1_ is the trend in referrals before the EHR lead-in; β_2_ is the change in referrals immediately following the EHR lead-in; β_3_ shows the trend in referrals after the EHR lead-in; β_4_ is the change in referrals immediately following the start of the provider education intervention; β_5_ shows the trend in referrals after the provider education intervention; and *e*_t_ represents the random variance. In time series data, there is a possibility that observations are correlated due to the proximity of their occurrence. Linear regression analysis assumes uncorrelated error terms between each outcome measurement. To prevent violating this assumption of linear regression and possibly miscalculating the confidence intervals, the Durbin-Watson test for auto-correlation was used to identify the presence of autocorrelation of successive error terms. Generally, a Durbin-Watson statistic close to 2 indicates less autocorrelation. We identified autocorrelation in our sample so the Prais-Winsten method was used to estimate the regression, which adjusts for autocorrelation (24). All analyses were conducted using STATA version 13 (StataCorp LP). Significance was established at *P* < .05.

**Figure:**
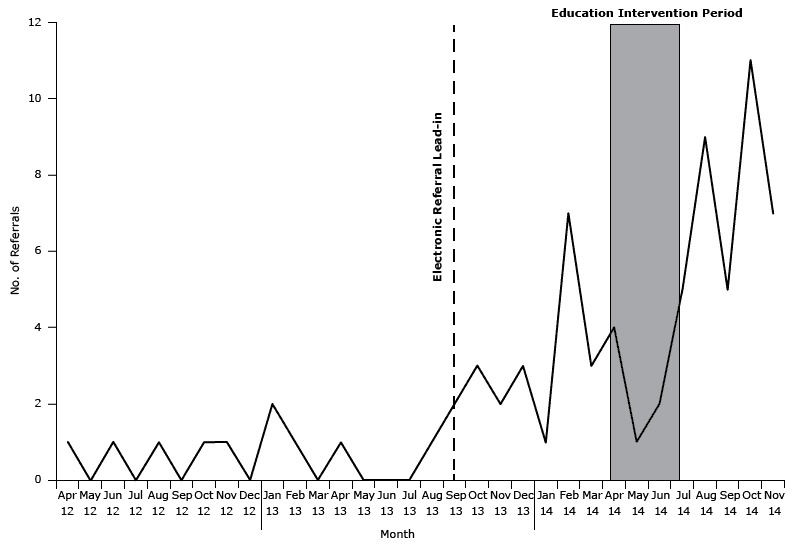
Patient referrals to YMCA Diabetes Prevention Program from April 2012 through November 2014 from 6 Federally Qualified Health Centers in the Bronx, New York. **Table:** 

Month	No. of Referrals
April 2012	1
May 2012	0
June 2012	1
July 2012	0
August 2012	1
September 2012	0
October 2012	1
November 2012	1
December 2012	0
January 2013	2
February 2013	1
March 2013	0
April 2013	1
May 2013	0
June 2013	0
July 2013	0
August 2013	1
September 2013	2
October 2013	3
November 2013	2
December 2013	3
January 2014	1
February 2014	7
March 2014	3
April 2014	4
May 2014	1
June 2014	2
July 2014	5
August 2014	9
September 2014	5
October 2014	11
November 2014	7

## Results

There were 75 referrals made in the 6 CATCH FQHCs from April 2012 through November 2014. The average age of the patients referred was 52 years, and most were female (70%). The average number of referrals was 1 per month before the electronic referral system lead-in and 3 patients referred per month after the electronic referral system lead-in on September 2013. Referrals increased to an average of 6 per month between the staff education intervention start in April and the end of the study period in November 2014 (Figure).

Segmented regression analysis (Table) shows that the trend in referrals before the electronic referral lead-in (β_1_[PreSlope1_t_]) and the change in levels of referrals around the point of the electronic referral system lead-in (β_2_[Int1_t_]) were not significant. However, there was a significant increasing trend of referrals after the EHR lead-in period (β_3_[PostSlope1t]). At the beginning of the provider education intervention (β_4_[Int2_t_]) there was a significant drop in referrals. There was also a significant positive trend in referrals after the provider education intervention (β_5_[PostSlope2_t_]) compared with before the intervention took place (β_3_[PostSlope1_t_]).

**Table:** Prais-Winsten Segmented Linear Regression Model Predicting Monthly YMCA Diabetes Prevention Program Referrals of Patients Seen During a Clinic Visit From April 2012 Through November 2015, Bronx, New York^a^

Independent Variable	Coefficient	Standard Error	*t* Statistic	*P* Value
Constant (β_0_)	0.656	0.406	1.61	.11
Pre trend (β_1_[PreSlope1_t_])	−0.009	0.039	−0.23	.81
**EHR referral lead-in**				
Change in level (β_2_[Int1_t_])	0.741	0.809	0.92	.36
Post trend (β_3_[PostSlope1_t_])	0.479	0.157	3.03	.006
**Provider education intervention**				
Change in level (β_4_[Int2_t_])	−4.53	0.899	−5.04	<.001
Post trend (β_5_[PostSlope2_t_])	0.731	0.195	3.75	.001

Abbreviation: EHR, electronic health record.

a Regression adjusted for autocorrelation based on Durbin-Watson statistic (original = 3.01; after transformation = 2.02).

## Discussion

The adaptation of DPP to community-based settings has met many challenges including the identification and enrollment of eligible participants (6,8,11,12). Our study was designed to 1) examine the effectiveness of referring patients with prediabetes to a community-based YDPP program through our FQHCs, and 2) determine whether referrals were sustained after the intervention. The results of our analyses show an increase in the number of eligible patients referred to the YDPP program after the provider education intervention with EHR lead-in. Whether the EHR lead-in contributed to the success of the provider education intervention is unclear. Some providers became aware of the electronic referral system and began using it before any official training on its use. We consider this a positive outcome of the electronic system change.

In these analyses, we chose to use total number of referrals as our outcome measure and not the rates of referral. We were more interested in whether provider referrals increased overall regardless of any changes in the overall number of the patient population eligible to be referred to YDPP. Nevertheless, we also did analyses using the proportion of referrals per eligible patients seen per month and the interpretation of the results remained the same (data not shown).

YDPP is perhaps the most evaluated adaptation of the DPP into community-based settings (8–12). Despite this adaptation, racial and ethnic minorities (21%) and low-income Medicaid recipients (6.7%) constitute a small percentage of those participating in New York State YDPPs (13). A low participation rate among racial and ethnic minorities and low-income patients indicates that work is needed to identify barriers to referral and participation in YDPP for these high-risk populations. The YDPP referral intervention evaluated in this study is part of many programs initiated under the Bronx CATCH collaboration. It is part of an ongoing effort to increase access to care and wellness in Bronx communities.

These data and the analyses performed have limitations. The study was not a randomized trial, and the quasi-experimental design used did not have a control group. We were unable to determine whether the apparent advantages of this intervention would have occurred because of some other phenomenon occurring simultaneously that would influence referrals (eg, a citywide push by the New York City Department of Health to increase awareness of prediabetes and treatment options). Furthermore, an effort began in September 2014 to meet with clinic medical providers, leadership, and administrators to review referral patterns, workflows, and patient results. This study did not fully capture the clinic-specific flow of information supporting the referral effort after the intervention meetings took place. The variability in referrals by clinic likely reflects differences in adaptation of the overall intervention and contributes to the rise and fall of referrals over the study period. The small number of referrals by clinic made analysis by clinic difficult in this preliminary investigation of the effect of our intervention on YDPP referrals. 

Clinic-to-community links may improve the quality of care for underserved patients (25). Connecting patients with neighborhood resources to address their health needs is a model consistent with the patient-centered medical home (http://www.pcmh.ahrq.gov/) and is aligned with the Affordable Care Act (www.hhs.gov/news/press/2014pres/08/20140826a.html). Referrals by health care providers are among the more successful strategies for identifying and enrolling eligible patients in YDPP (8), but for this strategy to be effective, new ways must be found to facilitate the provider referral process. The results of our study show promise for using the EHR system as a means of identifying and referring eligible patients to DPP programs. Medical directors of the target FQHCs felt prompted to act after being told that the clinic population included a high proportion of patients (approximately 33%) who met the criteria for prediabetes. They suggested that using the electronic system to quickly flag patients with prediabetes would help identify and refer more eligible patients. Furthermore, because women are more likely to participate in DPPs than are men (14,26,27), we believe that using the EHR to flag eligible patients can be a strategy to identify and refer more men to DPP.

Finally, on the basis of medical directors’ feedback, we are exploring strategies to develop a “warm hand-off” protocol with a more personalized referral, so the patients feel they have been introduced to YMCA staff that will provide the YDDP program. We will use a continuous quality improvement evaluation in our effort to increase the number of eligible patients who are referred and complete the YDPP. The quality improvement initiative will help us address strategies for sharing results with providers as a collaborative effort to improve patient care.
